# Supplementary material for: Risk assessment and clinical implications of COVID-19 in multiple myeloma patients: A systematic review and meta-analysis
Source: PLoS One. 2024 Sep 6;19(9):e0308463. doi: 10.1371/journal.pone.0308463 (PMC11379232; doi:10.1371/journal.pone.0308463)
Supplement: S2 Table — (DOCX) [file pone.0308463.s002.docx]

S2 Table: Search strings of keywords used in PubMed, Web of Science, and EMBASE databases

| **Search** | **Searching query** | **Articles** | **Date and time** |
| --- | --- | --- | --- |
| Pubmed | (((("multiple myeloma" [MeSH]) OR "multiple myeloma" [All Fields]) OR (("Multiple Myelomas" [MeSH]) OR "Multiple Myelomas" [All Fields]) OR (("Myelomatosis"[MeSH]) OR "Myelomatosis" [All Fields]) OR (("Myelomatoses"[MeSH]) OR "Myelomatoses" [All Fields]) OR (("Plasma Cell Myeloma"[MeSH]) OR "Plasma Cell Myeloma" [All Fields]) AND (((("COVID-19"[MeSH]) OR "COVID-19" [All Fields]) OR (("coronavirus" [MeSH]) OR "coronavirus" [All Fields]) OR (("2019 ncov"[MeSH]) OR "2019ncov" [All Fields]) OR (("wuhan"[MeSH]) OR "wuhan" [All Fields]) OR ("severe acute respiratory syndrome coronavirus 2" [All Fields]) OR (("SARS-CoV-2*"[MeSH]) OR "SARS-CoV-2*" [All Fields]) OR (("coronavirus 2019" [MeSH]) OR "coronavirus 2019" [All Fields]) OR (("nCoV disease"[MeSH]) OR "nCoV disease" [All Fields])))))  Date - Publication: January 1, 2020-April 12, 2024, Language: English | 474 | April 15, 2024; 11:13 pm |
| Web of Science | ((ALL=("multiple myeloma" OR "Multiple Myelomas" OR "Myelomas, Multiple" OR "Myelomatosis" OR "Myelomatoses" OR "Plasma Cell Myeloma" OR "Myeloma-Multiples")) AND ALL=("multiple myeloma" OR "Multiple Myelomas" OR "Myelomas, Multiple" OR "Myelomatosis" OR "Myelomatoses" OR "Plasma Cell Myeloma" OR "Myeloma-Multiples")) AND ALL=("COVID-19" OR "coronavirus" OR "2019ncov" OR "sars cov 2" OR "Wuhan" OR "severe acute respiratory syndrome coronavirus 2" OR "SARS-CoV-2" OR "nCoV disease" OR "2019-nCoV" OR "coronavirus 2019")  Date - Publication: January 1, 2020-April 12, 2024, Language: English | 582 | April 15, 2024; 11:13 pm |
| Embase | ('multiple myeloma'/exp OR 'multiple myeloma' OR 'multiple myelomas' OR 'myelomas, multiple' OR 'myelomatosis'/exp OR 'myelomatosis' OR 'myelomatoses' OR 'plasma cell myeloma'/exp OR 'plasma cell myeloma' OR 'myeloma-multiples') AND ('covid-19'/exp OR 'covid-19' OR 'coronavirus'/exp OR 'coronavirus' OR '2019ncov' OR 'sars cov 2'/exp OR 'sars cov 2' OR 'wuhan'/exp OR 'wuhan' OR 'severe acute respiratory syndrome coronavirus 2'/exp OR 'severe acute respiratory syndrome coronavirus 2' OR 'sars-cov-2'/exp OR 'sars-cov-2' OR 'ncov disease' OR '2019-ncov'/exp OR '2019-ncov' OR 'coronavirus 2019')  Date - Publication: January 1, 2020-April 12, 2024, Language: English | 2069 | April 16, 2024; 10:13 pm |
