# Supplementary material for: Risk assessment and clinical implications of COVID-19 in multiple myeloma patients: A systematic review and meta-analysis
Source: PLoS One. 2024 Sep 6;19(9):e0308463. doi: 10.1371/journal.pone.0308463 (PMC11379232; doi:10.1371/journal.pone.0308463)
Supplement: S3 Table — (DOCX) [file pone.0308463.s003.docx]

S3 Table : Risk of bias and quality assessment of selected studies using JBI critical appraisal checklist

| **JBI checklist for Cohort study** | **Wang et al.** | **Chari et al.** | **Hultcrantz et al.** | **Ho et al.** | **Krejci et al.** | **Ehsan et al.** | **Silfverberg et al.** | **Jin et al.** | **Karadeniz et al.** | **Musto et al.** | **Radoch et al.** | **Garnica et al.** | **JBI checklist for case series study** | **Martínez-López et al.** | **Susek et al.** |
| --- | --- | --- | --- | --- | --- | --- | --- | --- | --- | --- | --- | --- | --- | --- | --- |
| Were the two groups similar and recruited from the same population? | 1 | 1 | 1 | 1 | 1 | 0 | 0 | 1 | 0 | 1 | 0 | 1 | Were therSe clear criteria for inclusion in the case series? | 1 | 1 |
| Were the exposures measured similarly to assign people to both exposed and unexposed groups? | 1 | 0 | 1 | 1 | 1 | 1 | 1 | 1 | 1 | 1 | 1 | 1 | Was the condition measured in a standard, reliable way for all participants included in the case series? | 1 | 1 |
| Was the exposure measured in a valid and reliable way? | 1 | 1 | 1 | 1 | 1 | 1 | 1 | 1 | 1 | 1 | 1 | 1 | Were valid methods used for identification of the condition for all participants included in the case series? | 1 | 1 |
| Were confounding factors identified? | 0 | 0 | 0 | 0 | 0 | 1 | 0 | 0 | 0 | 0 | 0 | 0 | Did the case series have consecutive inclusion of participants? | 1 | 1 |
| Were strategies to deal with confounding factors stated? | 0 | 0 | 0 | 0 | 0 | 1 | 0 | 0 | 0 | 0 | 0 | 0 | Did the case series have complete inclusion of participants? | 1 | 1 |
| Were the groups/participants free of the outcome at the start of the study (or at the moment of exposure)? | 1 | 1 | 1 | 1 | 1 | 1 | 1 | 1 | 1 | 1 | 1 | 1 | Was there clear reporting of the demographics of the participants in the study? | 1 | 1 |
| Were the outcomes measured in a valid and reliable way? | 1 | 1 | 1 | 1 | 1 | 1 | 1 | 1 | 1 | 1 | 1 | 1 | Was there clear reporting of clinical information of the participants? | 1 | 1 |
| Was the follow up time reported and sufficient to be long enough for outcomes to occur? | 1 | 1 | 1 | 1 | 1 | 1 | 1 | 1 | 1 | 1 | 1 | 1 | Were the outcomes or follow up results of cases clearly reported? | 1 | 1 |
| Was follow up complete, and if not, were the reasons to loss to follow up described and explored? | 1 | 1 | 1 | 1 | 1 | 1 | 1 | 1 | 1 | 1 | 1 | 1 | Was there clear reporting of the presenting site(s)/clinic(s) demographic information? | 1 | 0 |
| Were strategies to address incomplete follow up utilized? | 0 | 0 | 0 | 0 | 0 | 0 | 0 | 0 | 0 | 0 | 0 | 0 | Was statistical analysis appropriate? | 1 | 1 |
| Was appropriate statistical analysis used? | 1 | 1 | 1 | 1 | 1 | 1 | 1 | 1 | 1 | 1 | 0 | 1 | NA | NA | NA |
| **Total score** | 8 | 7 | 8 | 8 | 8 | 9 | 7 | 8 | 7 | 8 | 6 | 8 |  | 10 | 9 |

NA- Not applicable
