# Supplementary material for: Risk assessment and clinical implications of COVID-19 in multiple myeloma patients: A systematic review and meta-analysis
Source: PLoS One. 2024 Sep 6;19(9):e0308463. doi: 10.1371/journal.pone.0308463 (PMC11379232; doi:10.1371/journal.pone.0308463)

S4 Fig 1: Forest plot from subgroup analysis by study type for hospitalization rate among patients with COVID-19 and multiple myeloma based on a random-effects model


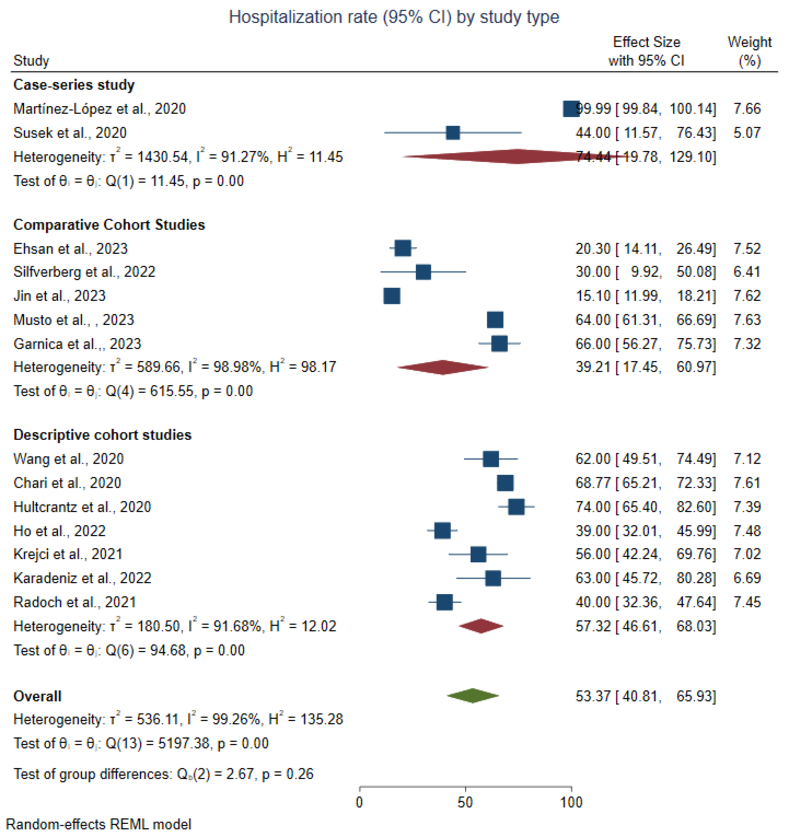


S4 Fig 2: Forest plot from subgroup analysis by study type for ICU admission rate among patients with COVID-19 and multiple myeloma based on a random-effects model


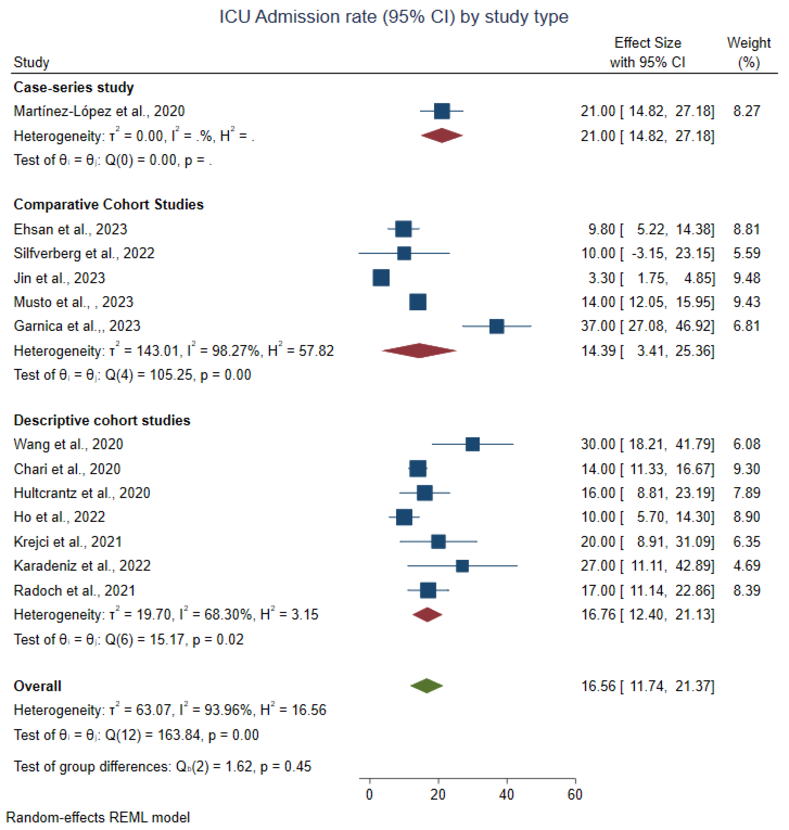


S4 Fig 3: Forest plot from subgroup analysis by study type for mortality rate among patients with COVID-19 and multiple myeloma based on a random-effects model


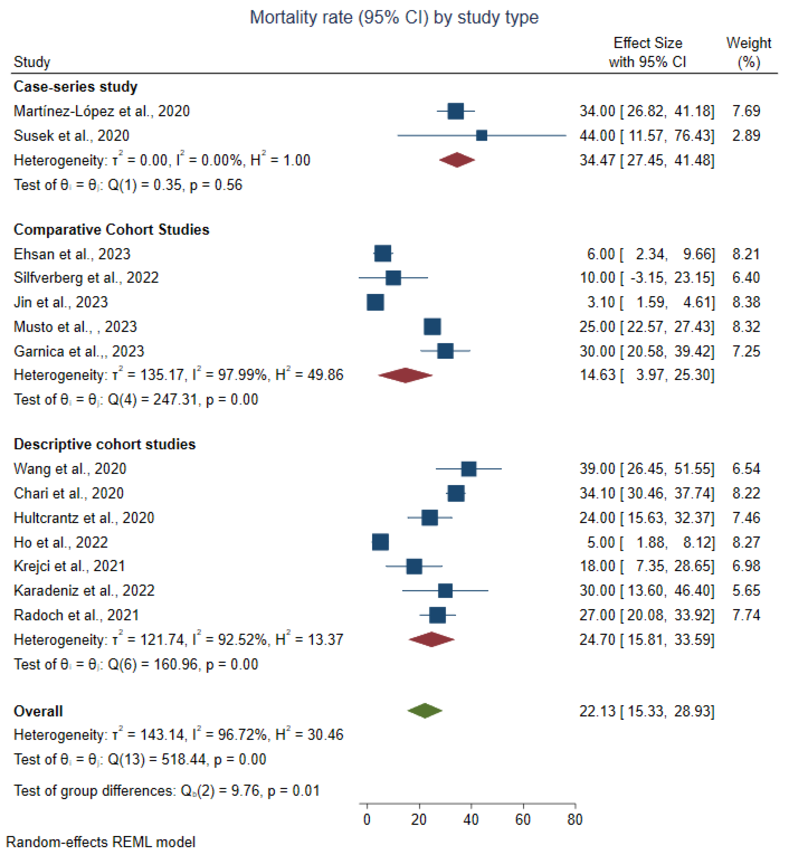


S4 Fig 4: Forest plot from subgroup analysis by study type for survival rate among patients with COVID-19 and multiple myeloma based on a random-effects model


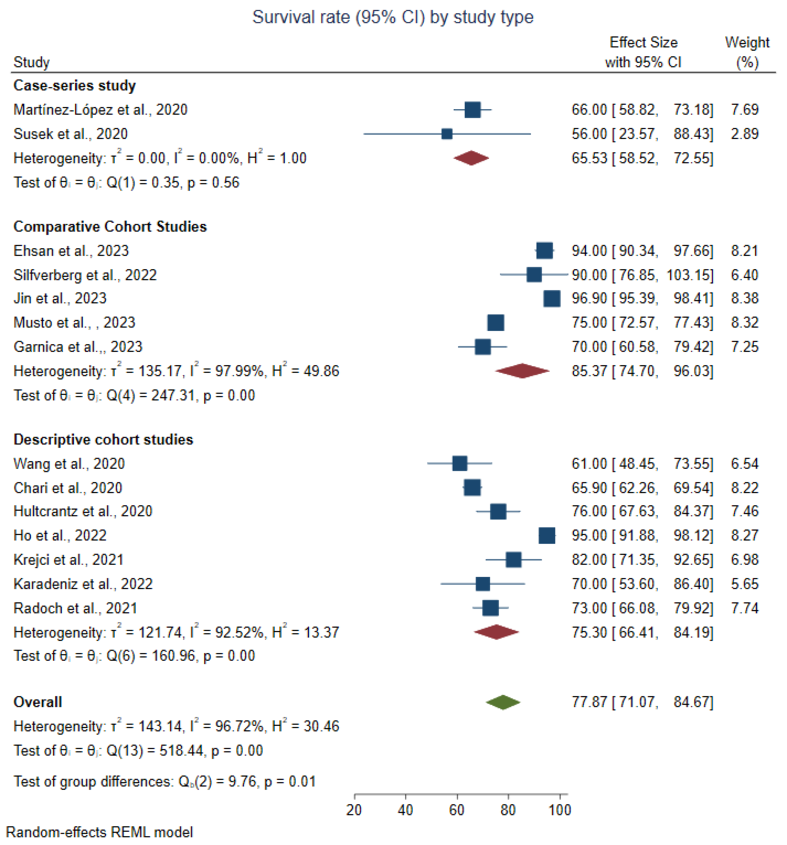


S4 Fig 5: Forest plot from subgroup analysis by sample size for hospitalization rate among patients with COVID-19 and multiple myeloma based on a random-effects model


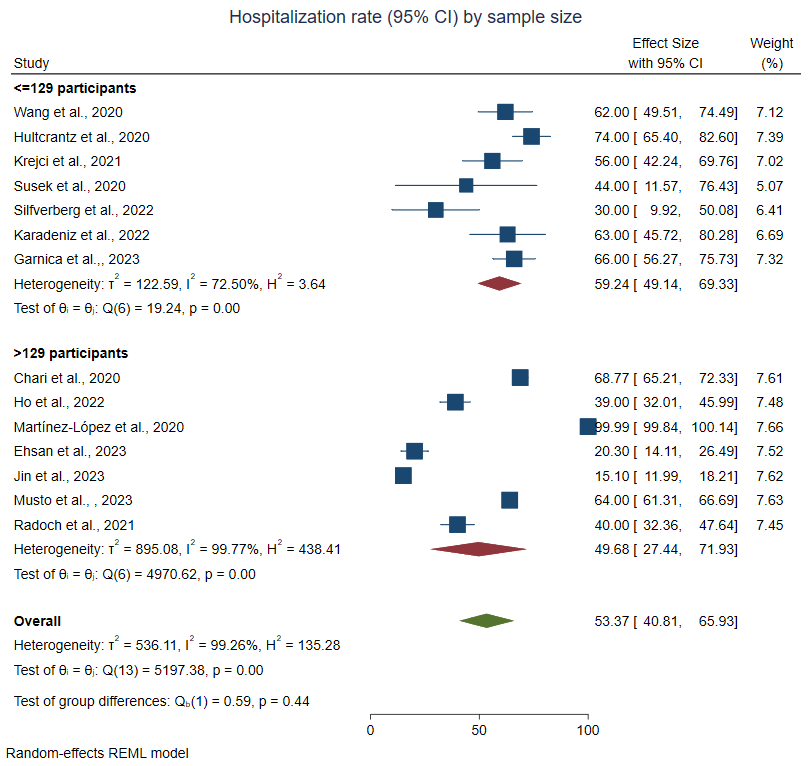


S4 Fig 6: Forest plot from subgroup analysis by sample size for ICU admission rate among patients with COVID-19 and multiple myeloma based on a random-effects model


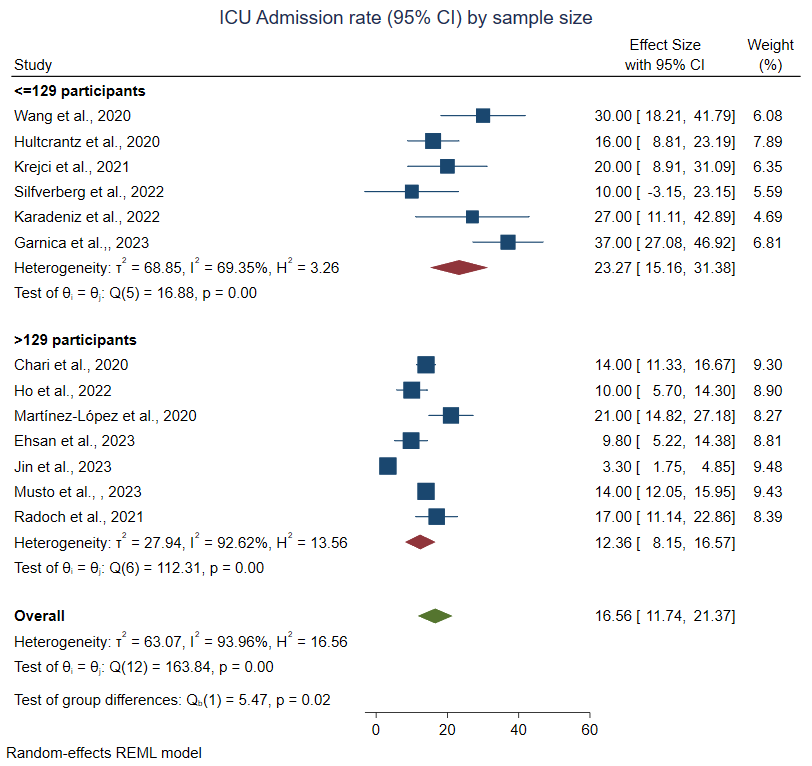


S4 Fig 7: Forest plot from subgroup analysis by sample size for mortality rate among patients with COVID-19 and multiple myeloma based on a random-effects model


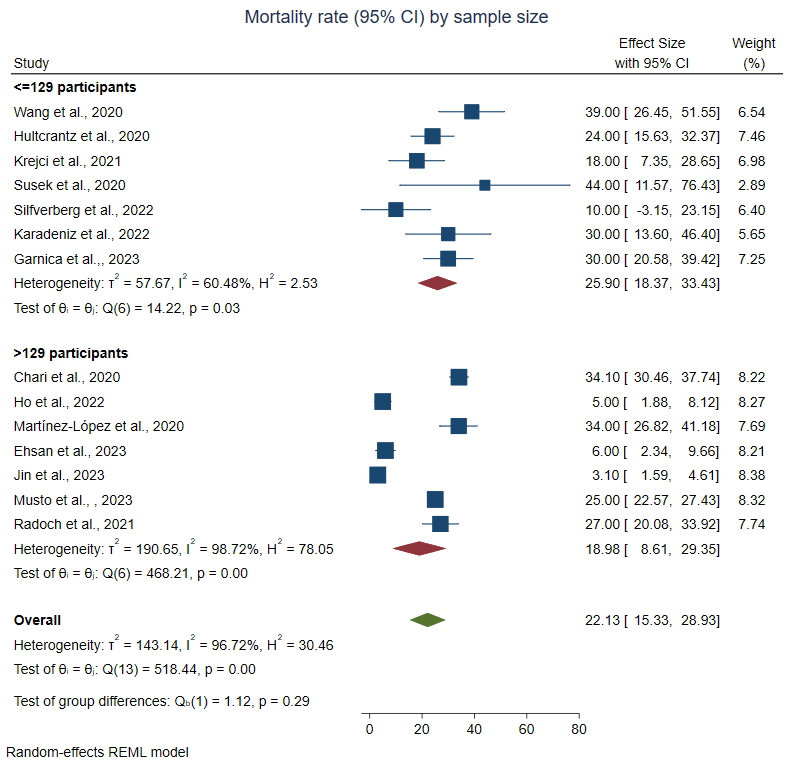


S4 Fig 8: Forest plot from subgroup analysis by sample size for survival rate among patients with COVID-19 and multiple myeloma based on a random-effects model


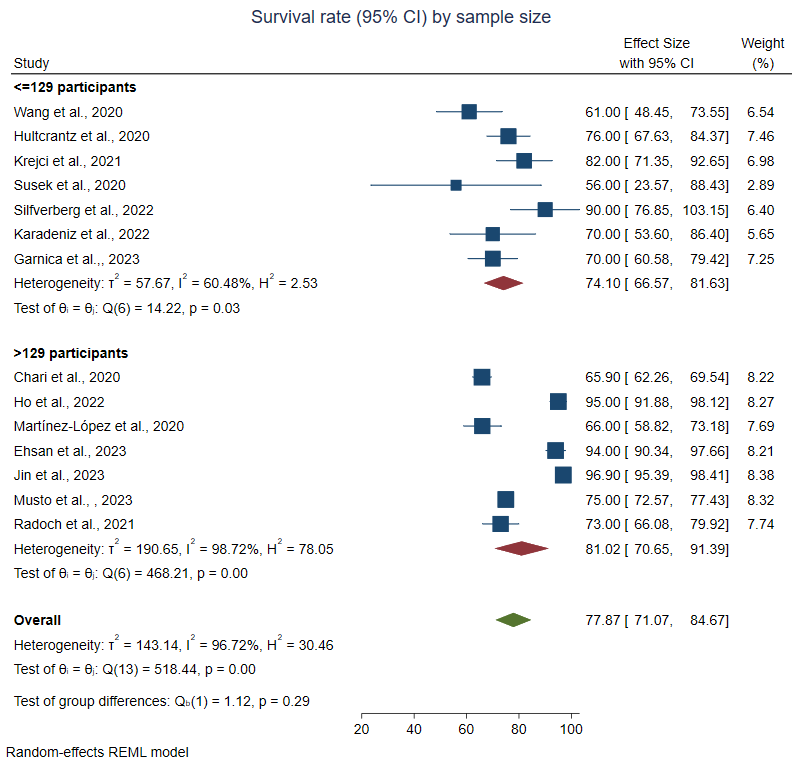


S4 Fig 9: Forest plot from subgroup analysis by median age for hospitalization rate among patients with COVID-19 and multiple myeloma based on a random-effects model


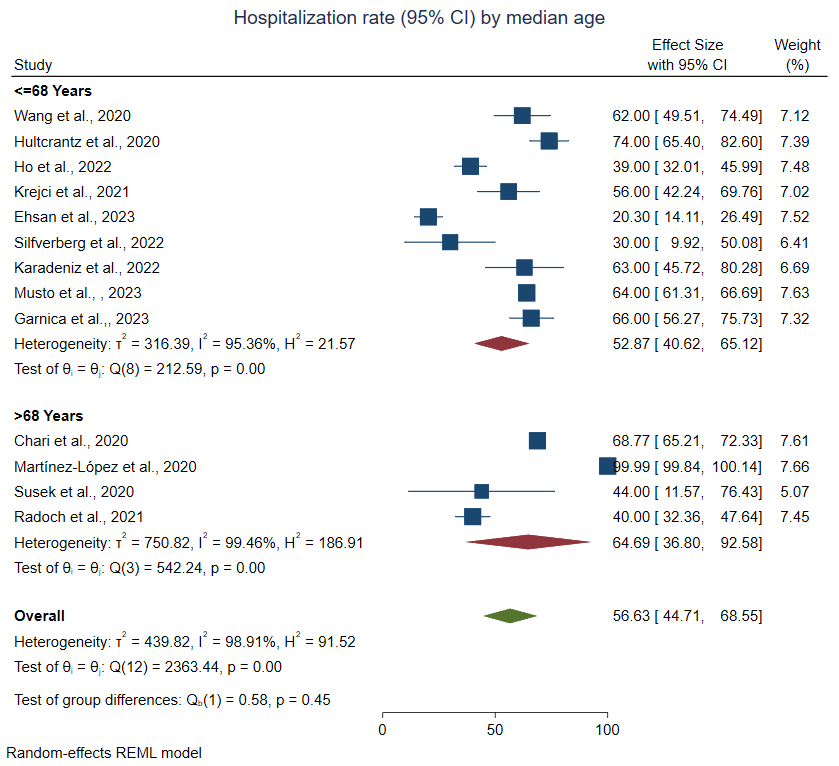


S4 Fig 10: Forest plot from subgroup analysis by median age for ICU admission rate among patients with COVID-19 and multiple myeloma based on a random-effects model


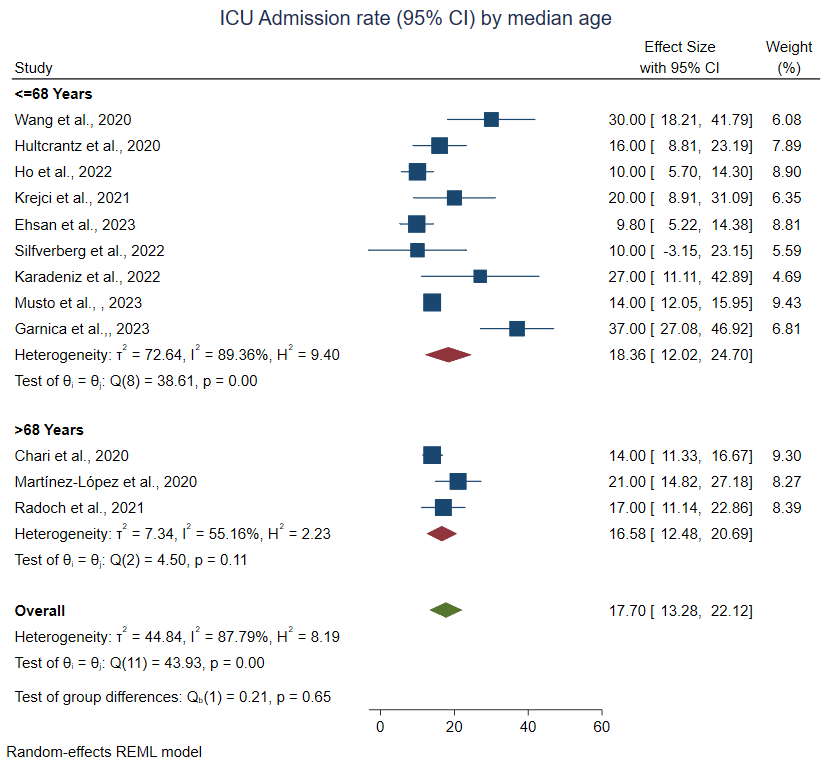


S4 Fig 11: Forest plot from subgroup analysis by median age for mortality rate among patients with COVID-19 and multiple myeloma based on a random-effects model


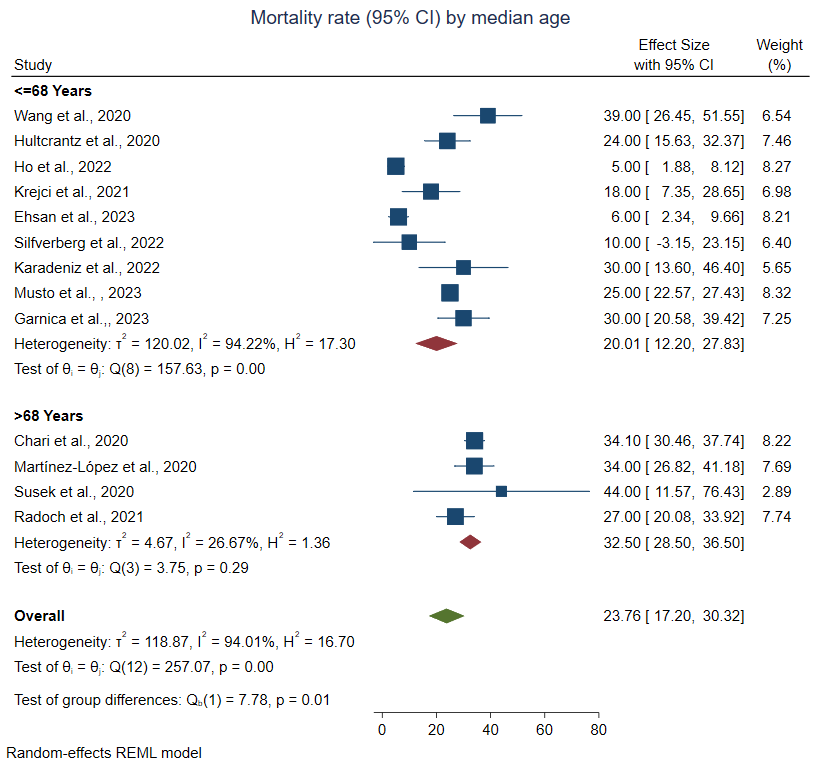


S4 Fig 12: Forest plot from subgroup analysis by median age for survival rate among patients with COVID-19 and multiple myeloma based on a random-effects model


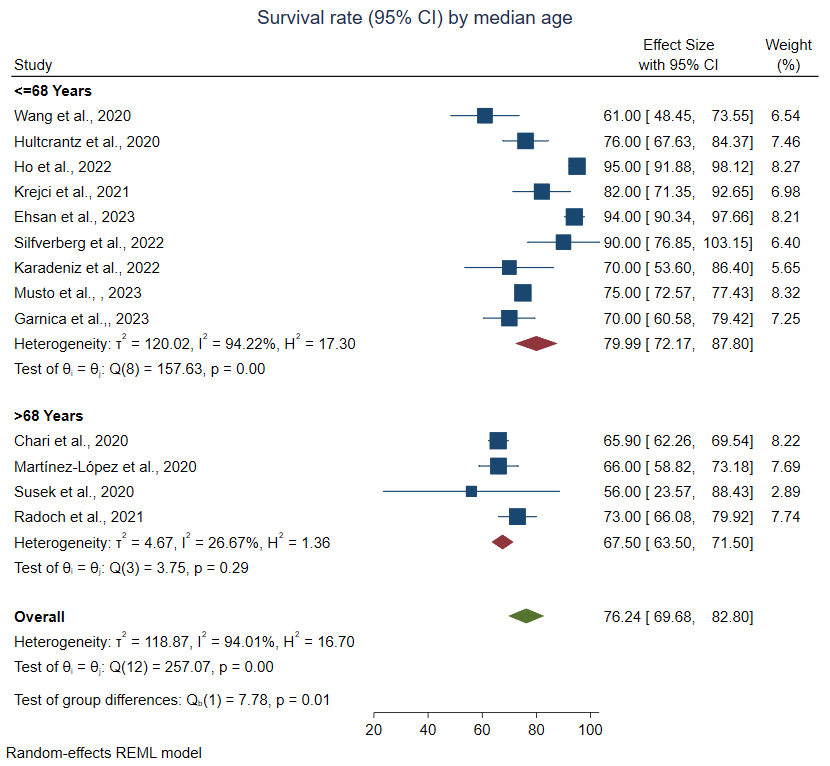


S4 Fig 13: Forest plot from subgroup analysis by proportion of men for hospitalization rate among patients with COVID-19 and multiple myeloma based on a random-effects model


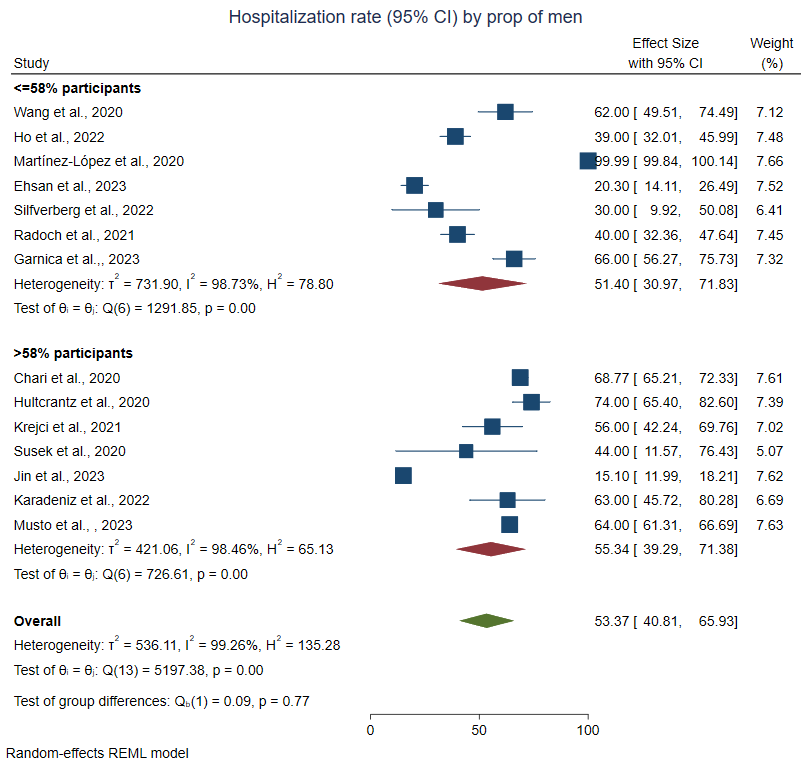


S4 Fig 14: Forest plot from subgroup analysis by proportion of men for ICU admission rate among patients with COVID-19 and multiple myeloma based on a random-effects model


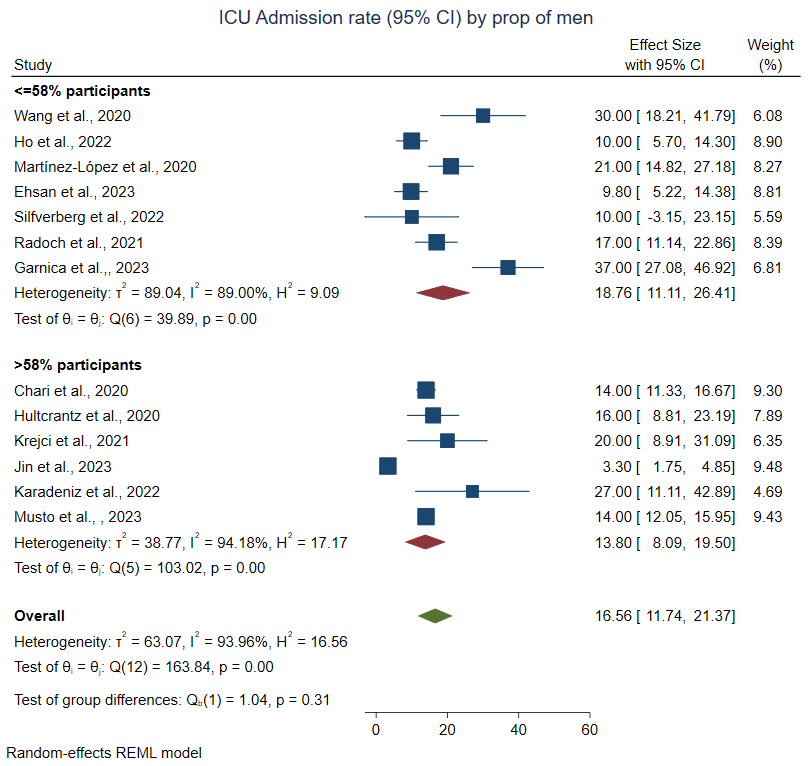


S4 Fig 15: Forest plot from subgroup analysis by proportion of men for mortality rate among patients with COVID-19 and multiple myeloma based on a random-effects model


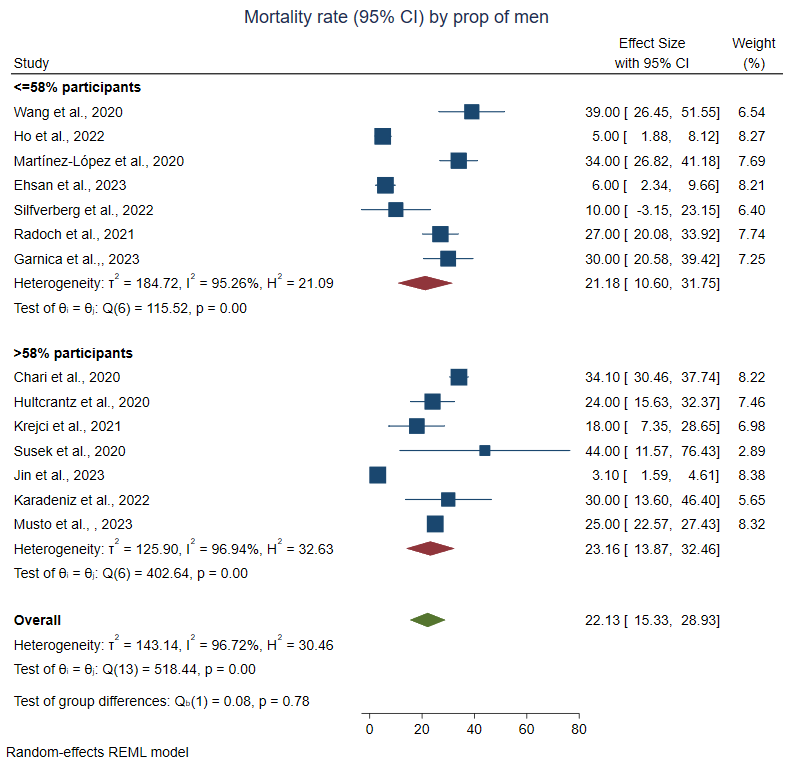


S4 Fig 16: Forest plot from subgroup analysis by proportion of men for survival rate among patients with COVID-19 and multiple myeloma based on a random-effects model


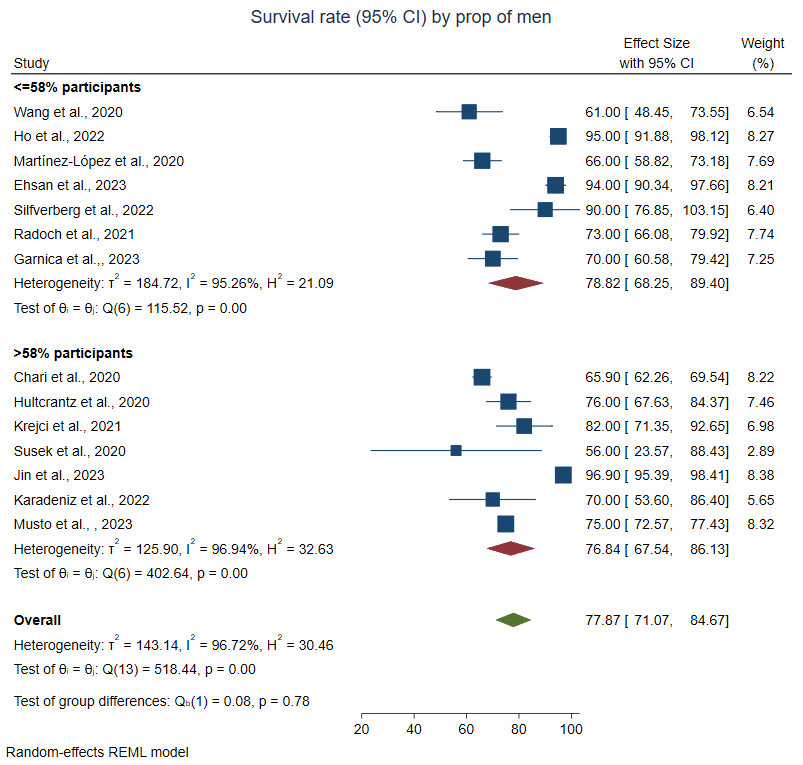

Supplement: S1 File — (DOCX) [file pone.0308463.s004.docx]
