# Supplementary material for: Risk assessment and clinical implications of COVID-19 in multiple myeloma patients: A systematic review and meta-analysis
Source: PLoS One. 2024 Sep 6;19(9):e0308463. doi: 10.1371/journal.pone.0308463 (PMC11379232; doi:10.1371/journal.pone.0308463)
Supplement: S1 Protocol — (PDF) [file pone.0308463.s005.pdf]

Assessing the impact of COVID-19 on multiple myeloma patients: a systematic review and meta-analysis

Review methods were amended after registration. Please see the revision notes and previous versions for detail.

Citation

Sultan Mahmud, MD Mohsin, Md Faruk Hossain, Ariful Islam, Harun Mazumder. Assessing the impact of COVID-19 on multiple myeloma patients: a systematic review and meta-analysis. PROSPERO 2023 CRD42023407784 Available from: https://www.crd.york.ac.uk/prospero/display_record.php?ID=CRD42023407784

Review question

What is the impact of COVID-19 on multiple myeloma patients, in terms of morbidity, mortality, and treatment outcomes?

Searches [1 change]

To conduct this systematic review and meta-analysis, a literature search will be performed between March 15 to 18, 2023, with a publication date limit of January 1, 2020, to March 10, 2023. The search queries will be designed using Boolean operators ("and," "or") and Medical Subject Headings (MeSH) terms for major databases, including "multiple myeloma", "Multiple Myelomas", "Myelomas, Multiple", "Myelomatosis", "Myelomatoses", "Plasma Cell Myeloma", "Myeloma-Multiples", "COVID-19", "coronavirus", "2019ncov", "sars cov 2", "Wuhan", "severe acute respiratory syndrome coronavirus 2", "SARS-CoV-2", "nCoV disease", "2019-nCoV", and "coronavirus 2019". The databases searched will be PubMed, EMBASE, Scopus, Global Health, and Web of Science, and relevant review articles and selected papers will also be examined for their bibliographies.

Types of study to be included

All types of observational studies will be included.

Studies meeting at least one of the following criteria will be excluded:

• Studies which did not measure the impact of COVID-19

• Studies did not present data on at least one outcome: mortality rate, survival rate, hospital admission rate, ICU admission rate

• Studies looking at vaccination

• Studies in which patients with other types of cancers

• Review articles and publications without original data (e.g., expert opinions, consensus statements, editorials)

• Studies published in a language other than English

• Animal studies.

Condition or domain being studied

Multiple myeloma patients infected with COVID-19.

Participants/population

Patients with diagnosed multiple myeloma and infected with the COVID-19.

Intervention(s), exposure(s)

This study will measure the effects of COVID-19 infection on multiple myeloma patients, including disease severity, hospitalization rates, treatment response, and overall survival rates.

Comparator(s)/control

Healthy individuals (patients without any malignancies who were listed as controls).

Main outcome(s)

The main outcomes of interest for this systematic review and meta-analysis are the impact of COVID-19 on multiple myeloma patients, including morbidity, mortality, and treatment outcomes. Specifically, the review aims to assess the following outcomes:

The severity of COVID-19 infection in multiple myeloma patients, including hospitalization rates, ICU admission rates, and need for mechanical ventilation.

Mortality rates associated with COVID-19 infection in multiple myeloma patients.

Impact of COVID-19 infection on multiple myeloma treatment outcomes, including treatment response rates, treatment delays or modifications, and overall survival rates.

Factors associated with increased risk of COVID-19 infection, severity, and mortality in multiple myeloma patients, such as age, comorbidities, and treatment regimens.

Additional outcome(s)

None.

Data extraction (selection and coding)

The data extraction for this systematic review and meta-analysis will involve a standardized approach to ensure consistency and accuracy across studies. The following data points will be extracted from each included study:

Study characteristics: including study design, location, setting, sample size, and duration of follow-up.

Patient characteristics: including age, sex, race/ethnicity, multiple myeloma diagnosis, disease stage, and comorbidities.

Intervention/exposure: including COVID-19 infection status, vaccine status, and treatment regimens for multiple myeloma.

Outcomes of interest: including incidence and prevalence of COVID-19 infection, severity of infection, mortality rates, treatment outcomes, and quality of life measures.

Statistical analysis: including summary measures, confidence intervals, and p-values.

The data extraction will be conducted by two independent reviewers, with any discrepancies resolved through discussion or consultation with a third reviewer if necessary. Data will be entered into a standardized data extraction form.

Risk of bias (quality) assessment

The risk of bias (quality) assessment will be carried out for all papers selected for inclusion in the systematic review and meta-analysis through the critical appraisal checklist for the qualitative research provided by the Joanna Briggs Institute (JBI).

Strategy for data synthesis

The strategy for data synthesis in this systematic review and meta-analysis will involve a comprehensive analysis of the data extracted from the included studies. The analysis will be conducted using appropriate statistical methods, with the aim of providing a summary estimate of the impact of COVID-19 on multiple myeloma patients.

Firstly, a narrative synthesis will be conducted to summarize the findings of the included studies and to identify any patterns or trends across the studies. This will involve a qualitative assessment of the study findings, including a discussion of the strengths and limitations of the studies.

Secondly, the meta-analyses for hospital admission rate, ICU admission rate, mortality rate, and survival rate will be carried out using the statistical software STATA 17. The pooled rates with 95% confidence interval will be estimated. Random-effects or fixed-effect models will be used depending on the heterogeneity of the studies, and subgroup analyses will be conducted to explore any potential sources of heterogeneity, such as study design, patient characteristics, or geographic location. All results in meta-analyses will be presented in forest plots.

Publication bias will also be assessed using appropriate statistical methods, such as funnel plots or Egger's regression test, and sensitivity analyses will be conducted to evaluate the robustness of the meta-analysis results to different assumptions or exclusions of studies.

Overall, the data synthesis strategy will be designed to provide a comprehensive and robust summary of the impact of COVID-19 on multiple myeloma patients and to identify any gaps in the literature or areas for further research.

Analysis of subgroups or subsets

Study design, study duration, sample size, age, sex, etc.

Contact details for further information

Sultan Mahmud

smahmud@isrt.ac.bd

Organisational affiliation of the review

International Centre for Diarrhoeal Disease Research, Bangladesh

Review team members and their organisational affiliations [1 change]

Mr Sultan Mahmud. International Centre for Diarrhoeal Disease Research, Bangladesh

Mr MD Mohsin. Institute of Statistical Research & Training (ISRT)

Md Faruk Hossain. Institute of Statistical Research & Training (ISRT)

Ariful Islam. University of Dhaka

Harun Mazumder. Institute of Statistical Research & Training (ISRT)

Type and method of review

Epidemiologic, Meta-analysis, Systematic review

Anticipated or actual start date

15 March 2023

Anticipated completion date

15 April 2023

Funding sources/sponsors

Undecided

Conflicts of interest

Language

English

Country

Bangladesh

Stage of review [1 change]

Review Completed not published

Subject index terms status

Subject indexing assigned by CRD

Subject index terms

COVID-19; Humans; Morbidity; Multiple Myeloma; Patients; Treatment Outcome

Date of registration in PROSPERO

14 March 2023

Date of first submission

13 March 2023

Stage of review at time of this submission [3 changes]

Preliminary searches	Yes	Yes
Piloting of the study selection process	Yes	Yes
Formal screening of search results against eligibility criteria	Yes	Yes
Data extraction	Yes	Yes
Risk of bias (quality) assessment	Yes	Yes
Data analysis	Yes	Yes

Revision note

I have marked the review status as completed but unpublished and included a new member who played an active role in the review process.

The record owner confirms that the information they have supplied for this submission is accurate and complete and they understand that deliberate provision of inaccurate information or omission of data may be construed as scientific misconduct.

The record owner confirms that they will update the status of the review when it is completed and will add publication details in due course.

Versions

14 March 2023

18 March 2023

27 March 2023

18 April 2023
