# Supplementary material for: Risk assessment and clinical implications of COVID-19 in multiple myeloma patients: A systematic review and meta-analysis
Source: PLoS One. 2024 Sep 6;19(9):e0308463. doi: 10.1371/journal.pone.0308463 (PMC11379232; doi:10.1371/journal.pone.0308463)
Supplement: Attachment — Submitted filename: Response to Reviewers.docx [file pone.0308463.s006.docx]

June 21, 2024

Emily Chenette

Editor-in-chief

PLOS ONE

Manuscript Number: PONE-D-24-17495

Title: Risk Assessment and Clinical Implications of COVID-19 in Multiple Myeloma Patients: A Systematic Review and Meta-Analysis

Dear Editor,

Thank you for allowing us to resubmit the revised manuscript. We appreciate the time and effort that you and the reviewers have dedicated to providing us with your valuable feedback on our manuscript. We are truly grateful to the reviewers for their insightful comments on our paper. We believe these suggestions will help improve the quality of the manuscript. We have been able to incorporate changes to reflect all the suggestions provided by the reviewers. We have used track changes option within the manuscript to highlight the changes.

Here is the point by point responses to the reviewers’ comments and concerns:

**Response to Reviewer #1:**

**Reviewer point #1:** Why are there up to three references for PRISMA? How do the first two constitute PRISMA?

**Author response #1:**

*Thank you for catching this. We have removed these irrelevant citations from the revised manuscripts (page #2).*

**Reviewer point #2:** Can the authors briefly describe the JBI scoring under the quality assessment section?

**Author response #2:**

*Thank you for your valuable suggestion. We have added more description of the JBI scoring in both the Methods section (pages 3-4) and the Results section (page 8). During the update, we noticed some discrepancies in the JBI checklist. Therefore, we rescored all the studies using the most appropriate and updated checklist (see Supporting Information S3.).*

**Reviewer point #3:** Consider doing a leave-one-out sensitivity analysis to report the influence of each study on the pooled prevalence rates for your major outcomes.

**Author response #3:**

*Thank you for your insightful suggestion. We appreciate your recommendation to perform a leave-one-out sensitivity analysis to assess the influence of each study on the pooled prevalence rates for our major outcomes. This analysis will provide valuable insights into the robustness and stability of our findings.*

*We have conducted the leave-one-out sensitivity analysis as suggested. Both the Method (Page # 3) and Result section (Page# 9, Figure 4) has been updated accordingly.*

**Reviewer point #4:** Table 3 can be taken to the supplementary file.

**Author response #4:**

*Yes, we have added Table 3 as suppliementary information (S3 file).*

**Reviewer point #5:** The authors classified the available included studies into three: case series, retrospective cohort studies, and “retrospective observational studies” Can the authors define what they mean by “retrospective observational studies” since even cohort studies are also observational and can be retrospective (as in much of your included studies) or prospective, just as case series are also a type of observational studies.

**Author response #5:**

*Thank you for your insightful comment. Yes, we completely agree with you the terminology is confusing as cohort studies are also observational by nature. To avoid confusion, we have revised our terminology in the revised manuscript to:*

- *Case Series*
- *Comparative Cohort Studies*
- *Descriptive Cohort Studies.*

*We have also added clear definitions of each term in the Method section (Page#4-5) as follows:*

- ***Case Series****: Studies that present descriptive analysis of cases with a common characteristic, lacking a comparative group.*
- ***Comparative Cohort Studies****: These studies identify a cohort (group) of individuals who share a common characteristic or exposure in the past and then look back to compare outcomes between subgroups within this cohort. They typically include a comparison group and allow for some measure of association between exposure and outcome.*
- ***Descriptive Cohort Studies****: These studies analyze existing data without the formal structure of a cohort study. They often analyze data from medical records or databases to identify patterns, outcomes, and associations. Descriptive cohort studies do not involve comparing outcomes between different subgroups within the cohort.*

*Please note that, based on the new categorization, all analyses by study type have been updated.*

**Reviewer point #6:** Please, note that Egger’s test figures are missing from the main manuscript text and supporting files. Please, reference each figure when you include them in your revision.

**Author response # 6:**

*We have added a Table (Table 3) in revised manuscript (Page# 11), presenting the Egger test results.*

**Reviewer point #7:** Why have the authors not considered doing a meta-analysis on the risk factors for each of their four main outcomes (COVID-19-related hospitalization rate, ICU admission rate, mortality rate, and survival rate), especially given the fact that you stated your study’s title as “risk assessment”? Aren’t there data for such analysis from the original studies?

**Author response #7:**

*Thank you for your feedback. We appreciate the suggestion to conduct a meta-analysis on the risk factors for each of our four main outcomes (COVID-19-related hospitalization rate, ICU admission rate, mortality rate, and survival rate).*

*While we agree that such an analysis would be valuable, our primary limitation was the lack of detailed data on risk factors across the included studies. Most of the original studies did not provide sufficient information on specific risk factors. However, we have conducted subgroup analyses for study-level characteristics such as median age, proportion of men, and patients' comorbidities (hypertension, diabetes, obesity), identifying factors that may influence the outcomes of hospitalization rates, ICU admissions, mortality rates, and survival rates.*

*In our manuscript, we aimed to highlight the available data on these outcomes and acknowledged the limitations posed by the variability and incompleteness of risk factor reporting (Page #15). We believe that addressing this gap is crucial for future research, and we have included a recommendation for more standardized and detailed reporting of risk factors in original studies in our discussion section (Page #15).*

**Response to Reviewer #2:**

**Reviewer point #1:** A great manuscript. Very comprehensive and most of analysis needed for a systematic review and meta analyses conducted well. Will possibly attract a lot of citations with the wealth of information that is being presented in this article.

**Author response #1:**

*Thank you very much for your positive feedback. We are delighted to hear that you found our manuscript comprehensive and well-conducted. We appreciate your kind words regarding the potential impact and value of our work.*

**Additional requirements:**

1. Please ensure that your manuscript meets PLOS ONE's style requirements, including those for file naming. The PLOS ONE style templates can be found at 
<https://journals.plos.org/plosone/s/file?id=wjVg/PLOSOne_formatting_sample_main_body.pdf> and 
<https://journals.plos.org/plosone/s/file?id=ba62/PLOSOne_formatting_sample_title_authors_affiliations.pdf>

2. Please amend the manuscript submission data (via Edit Submission) to include author Dr. Md Faruk Hossain.

3. Please amend your authorship list in your manuscript file to include author Dr. Sultan Faruk Mahmud.

**Author response #1:**

*Thank you very much for your suggestions. We have ensured that our manuscript meets PLOS ONE's style requirements, including file naming, as per the guidelines provided. The author list in the manuscript was correct; however, we have amended the manuscript submission data to fix the authorlist.*
